# Feature Identification Using Interpretability Machine Learning Predicting Risk Factors for Disease Severity of In-Patients with COVID-19 in South Florida

**DOI:** 10.3390/diagnostics14171866

**Published:** 2024-08-26

**Authors:** Debarshi Datta, Subhosit Ray, Laurie Martinez, David Newman, Safiya George Dalmida, Javad Hashemi, Candice Sareli, Paula Eckardt

**Affiliations:** 1 Christine E. Lynn College of Nursing, Florida Atlantic University, Boca Raton, FL 33431, USA; 2 College of Engineering & Computer Science, Florida Atlantic University, Boca Raton, FL 33431, USA; 3 Memorial Healthcare System, Hollywood, FL 33021, USA

**Keywords:** COVID-19 predictive model, random forest classifier, SHAP, Gini index, permutation-based interpretation, caring data science

## Abstract

**Objective:** The objective of the study was to establish an AI-driven decision support system by identifying the most important features in the severity of disease for **I**ntensive **C**are **U**nit (ICU) with **M**echanical **V**entilation (MV) requirement, ICU, and **I**nter**M**ediate **C**are **U**nit (IMCU) admission for hospitalized patients with COVID-19 in South Florida. The features implicated in the risk factors identified by the model interpretability can be used to forecast treatment plans faster before critical conditions exacerbate. **Methods:** We analyzed eHR data from 5371 patients diagnosed with COVID-19 from South Florida Memorial Healthcare Systems admitted between March 2020 and January 2021 to predict the need for ICU with MV, ICU, and IMCU admission. A Random Forest classifier was trained on patients’ data augmented by SMOTE, collected at hospital admission. We then compared the importance of features utilizing different model interpretability analyses, such as SHAP, MDI, and Permutation Importance. **Results:** The models for ICU with MV, ICU, and IMCU admission identified the following factors overlapping as the most important predictors among the three outcomes: age, race, sex, BMI, diarrhea, diabetes, hypertension, early stages of kidney disease, and pneumonia. It was observed that individuals over 65 years (‘older adults’), males, current smokers, and BMI classified as ‘overweight’ and ‘obese’ were at greater risk of severity of illness. The severity was intensified by the co-occurrence of two interacting features (e.g., diarrhea and diabetes). **Conclusions:** The top features identified by the models’ interpretability were from the ‘sociodemographic characteristics’, ‘pre-hospital comorbidities’, and ‘medications’ categories. However, ‘pre-hospital comorbidities’ played a vital role in different critical conditions. In addition to individual feature importance, the feature interactions also provide crucial information for predicting the most likely outcome of patients’ conditions when urgent treatment plans are needed during the surge of patients during the pandemic.

## 1. Introduction

The COVID-19 pandemic has significantly affected global health and economies, overwhelming hospitals and straining limited resources [1]. As of April 2024, there have been over 700 million confirmed cases of COVID-19 and over 7 million deaths worldwide, with Florida alone accounting for almost 1.14% of all confirmed cases and approximately 1.36% of worldwide deaths [2]. The immense strain on healthcare systems, particularly in South Florida, necessitated the development of a predictive model for **I**ntensive **C**are **U**nit (ICU) with **M**echanical **V**entilation (MV) requirement, ICU, or **I**nter**M**ediate **C**are **U**nit (IMCU) admission to effectively allocate resources, optimize patient care, and improve outcomes for patients with COVID-19. All patients who required MVs were admitted to the ICU; however, not all patients in the ICU necessarily needed MV. For ease of presentation, we shall refer to ICU patients with MV as only MV for brevity.

The COVID-19 pandemic has emphasized the critical need for optimizing resource allocation in healthcare systems. The demand for ICU beds surged due to COVID-19, with estimates of ICU admission rates ranging from 5% to 30% [3,4]. This increased the demand for critical care resources, including ventilators, posed challenges, and led to shortages during the peak of the pandemic [5]. Accurately predicting patients at a higher risk of requiring intensive care interventions allows healthcare providers to proactively allocate resources such as ICU beds, ventilators, and specialized staff to the patients who need them the most [6]. This targeted resource allocation ensures that critical care resources are utilized effectively and judiciously, maximizing their impact on patient outcomes.

In addition, predicting IMCU admission allows for proper triage and prevents the underutilization or overburdening of ICU resources. The early identification of patients requiring IMCU admission enables healthcare providers to intervene promptly and provide appropriate care. Patients in the IMCU may require specialized monitoring, non-invasive ventilation, or other interventions to manage their respiratory status or other critical care needs. Not all patients require the care provided in an ICU, but they may still benefit from closer monitoring and specialized interventions available in the IMCU. This improves care coordination, patient flow, and cost-effective resource management. It allows hospitals to anticipate the number of patients requiring intensive care, plan staffing and equipment needs, as well as coordinate care across different healthcare facilities. By adopting a proactive approach, healthcare systems can better manage an influx of patients, maintain quality care, and ensure that critical care is provided to those in need [7]. Timely, individualized interventions can also help prevent disease progression, reduce complications, and improve patient outcomes [8].

Given the demands for critical decisions in the healthcare system, clinicians can also benefit from AI-driven decision support systems for deciding optimal treatment plans for hospitalized patients with COVID-19 when there is an urgent need for decision making. AI-driven decision support systems can provide insights into disease severity, prognosis, and enable healthcare providers to better communicate disease diagnoses to patients and their families, make informed decisions about end-of-life care, and allocate resources appropriately based on the patient’s likelihood of recovery [9]. AI has been successfully deployed in clinical settings to aid in many healthcare decisions [10], such as detecting diseases [11], risk assessments [12], and personalized outcomes [13,14]. 

### 1.1. An Overview of Machine Learning Studies on COVID-19 Disease Severity

The literature on COVID-19 disease severity has undergone thorough analyses using various predictive methods. **M**achine **L**earning (ML) has been used to develop scoring tools based on essential features to measure COVID-19 disease severity [15,16,17] and to predict the disease severity scores of patients presented by a standardized severity scale, such as the National Early Warning Score 2 (NEWS2) [18]. Thus, ML tools can learn to predict outcomes based on a known severity metric or establish a new severity scale that improves the known risk assessment methods by incorporating novel features not included in the standardized scales [17]. Furthermore, past studies have focused on predicting the severity conditions of in-patients, such as MV requirements [18,19,20,21], days spent in ICU [20,21,22,23,24], whether rehospitalization is necessary for recurring health problems [15,25], and forecasting the need for other therapeutic interventions [24]. 

The studies have used various datasets to train their models; some relied solely on lab biomarkers [24,26], while others utilized medical histories like **e**lectronic **H**ealth **R**ecords (eHRs) and demographic information [21], and a few combined both data [19,23,25]. Additionally, some studies incorporated features from medical imaging, eHRs, and laboratory tests [22,27,28]. It is worth noting that the performance of the ML models reported in the studies varied considerably. Some achieved a high performance with an **A**rea **U**nder the **C**urve (AUC) exceeding 0.90, specifically in studies with smaller cohorts [27,29]. This could be potentially due to overfitting the specific population tested. For instance, Hong et al. [29] reported an accuracy of over 0.90 for ML prediction when trained on only 63 patients with COVID-19 to predict the severity of illness. Similarly, Liu et al. [27] achieved very high accuracy (AUC > 0.96) on a small patient cohort of approximately 158 individuals. Nevertheless, studies that involve larger cohorts of over 5000 patients [15,21,25] exhibit slightly lower accuracy (AUC < 0.90), but offer generalizability over a larger population. 

Furthermore, numerous studies on lab biomarkers have yielded notably robust accuracy. The same performance can also be achieved when the model has been trained on demographics and eHR data, suggesting that a viable alternative during a pandemic crisis when hospital resources are limited and obtaining lab test results become challenging. Multiple ML approaches have been employed, including boosting-based classifiers [18,24,25,26,27], regression methods [15,17,25,30], Support Vector Machine [18,24,27], **R**andom **F**orest (RF) classifier [18,24,27,30], and Naïve Bayes classifier [17,26]. In comparative studies assessing the accuracy of various ML models, it is worth noting that boosting methods, such as Catboost, and XGBoost, have been reported to deliver the highest accuracy, as documented by Noy et al. [18], Liu et al. [27] and Hong et al. [29]. Nevertheless, several other comparative studies [24,30] have indicated that an RF classifier outperforms other ML models. Consequently, our selection of classification methods remains competitive with previous studies. 

Additionally, investigations into **D**eep **L**earning (DL) neural network-based approaches [21,23] produced similar accuracy results to their ML counterparts and have also been shown to outperform in certain cases [23]. Furthermore, a hybrid approach has been reported combining DL and ML into a single predictive architecture, where the DL extracted features for chest CT scan images utilized by a CatBoost model downstream along with lab biomarkers and eHR data to predict COVID-19 disease severity with high accuracy were employed [22]. This allows for integrating radiological information with other laboratory and clinical reports, further enabling predictive models to make critical decisions [22].

Many studies have tried to discover the main contributing features to risk prediction utilizing statistical measures [17,20], coefficient scores of regression models [15,21,25,27,30], and other interpretability approaches, such as the Gini index of feature importance [15,23], permutation-based approaches, and SHAP (**SH**apley **A**dditive ex**P**lanations)-based feature analysis [22,26]. The frequently emphasized demographic factors in predicting COVID-19 disease severity are age, hypertension, diabetes, heart disease, gender, smoking status, and **C**hronic **K**idney **D**isease (CKD) [15,17,20,25]. 

It has been shown that the studies incorporating eHRs and lab tests tend to assign higher importance to lab biomarkers in their feature ranking as they are direct measures of current health conditions pertaining to disease severity [18,22,24]. However, studies that relied exclusively on eHR data also performed comparably, identifying comorbidities and other demographic variables as top-ranking features [20]. SHAP analysis can provide additional insights by emphasizing individual features with a more pronounced impact on severity [18]. For instance, it has been found that older age, lower platelet counts, and lower lymphocyte levels are closely associated with COVID-19 disease severity [18]. It is worth noting that the exploration of individual class-based SHAP scores for demographic data has been limited and mainly exists in studies focusing on lab biomarkers. Our current study presents SHAP scores for each feature that contributes to predicting MV requirement, ICU, or IMCU admission.

### 1.2. Contributions of the Current Study

Our current study focuses on a large South Florida cohort, a previously explored dataset for COVID-19 disease severity predictive analysis. Previously, Datta et al. [31] utilized the same dataset to find the most critical features underlying mortality risk, utilizing an RF classifier and SHAP-based interpretability. A comparative analysis utilizing the performance of traditional ML, DL, and the fine-tuned **L**arge **L**anguage **M**odel (LLM) in our previous study revealed that the RF model has the highest precision and accuracy compared to other traditional ML (e.g., XGBoost and KNN) or DL (e.g., MLP) models [32].

Here, we extended the work to understand the essential features indicative of COVID-19 disease severity for in-patients from South Florida for predicting therapeutic interventions, such as ICU with MV requirement, ICU, and IMCU admission. A similar severity prediction was performed using DL on the same dataset [33]; however, the severity scale was not well-defined. Furthermore, the analysis lacked the ability to interpret the model’s decision. The current study explores a better metric for patients, caregivers, and clinicians, which is a direct prediction of therapeutic interventions. Additionally, numerous studies focused solely on comparing ML models and did not examine different interpretability approaches for feature analysis. 

Thus, our contributions to this work are two-fold: we determine the severity of COVID-19 disease by utilizing the three distinct RF classifiers, and compare the importance of features across the cases susceptible to the conditions. This comparison shows how different features are similar across different severity cases and vary across the conditions.

To enhance the reliability of the findings, we utilized multiple interpretability methods to better understand the essential features, as their importance varies across many studies. In addition, we analyzed the interactions between SHAP features to understand how combinations of features impact the severity of COVID-19 disease.

## 2. Materials and Methods

### 2.1. Dataset Collection and Subject Information

With the exemption of informed consent and the HIPAA waiver, the **I**nstitutional **R**eview **B**oard (IRB) approved the study and is exempt from further review. From 14 March 2020 to 16 January 2021, data were obtained from the **M**emorial **H**ealthcare **S**ystem (MHS), Hollywood, FL, USA and investigated by the co-authors from the Christine E. Lynn College of Nursing and College of Engineering and Computer Science at Florida Atlantic University. 

For this project, retrospective data for 5371 hospitalized patients with confirmed cases of COVID-19, as defined by the RT-PCR sampling of nasal and pharyngeal swabs, were retrieved from a comprehensive healthcare system in South Florida. Initially, 203 (independent patient variables) contained data on ‘patients’ sociodemographic characteristics’ (e.g., age, sex, BMI, and smoking status), ‘pre-hospital comorbidities’ (e.g., diarrhea, diabetes, and pneumonia), and ‘medications’ (e.g., **A**ngiotensin **R**eceptor **B**lockers (ARBs) and **A**ngiotensin **C**onverting **E**nzyme (ACE) inhibitors) [31].

### 2.2. Study Design Considerations

**Figure 1:**
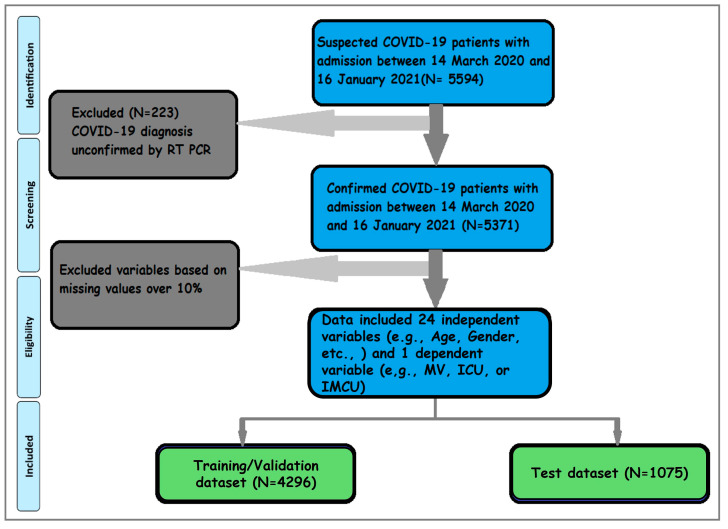
*Work flowchart*. The flowchart was modified from our previous study on the same dataset [31]. The data inclusion strategy is shown in the flowchart. Out of 5594 hospitalized cases, 5371 are confirmed cases with COVID-19. The inclusion criteria for 25 variables (24 independent and one of three dependent variables (ICU with MV, ICU, or IMCU)) are included in the study after data preprocessing.

Figure 1 presents the visual abstract of a complete data science cycle. The input dataset for our analysis consisted of 5594 patients admitted to the hospital with COVID-19-related symptoms. Among the 5371 patients with COVID-19, 4296 (80%) were in the training dataset, while the remaining 1075 (20%) were in the test dataset. Preprocessing steps ensured data quality by eliminating repeating variables and removing the features with over 10% missing values [34] from the patients’ eHRs. In the current study, only BMI had 6.5% missing values among the remaining independent variables, for which we utilized the Bayesian Ridge Imputation method [30]. Three separate models were trained utilizing 25 variables, comprising 24 independent variables for each dependent variable: MV, ICU, or IMCU.

### 2.3. Data Classification

The current research is based on binary classification problems with one of three dependent variables (MV, ICU, or IMCU). Each dependent variable was used in a different model and segmented into binary consequences, classified either as ‘MV requirement’ (‘1’) vs. ‘no MV requirement’ (‘0’) or ‘ICU admission’ (‘1’) vs. ‘no ICU admission’ (‘0’), or ‘IMCU admission’ (‘1’) vs. ‘no IMCU admission’ (‘0’). The labels were then converted to binary numerical values before training the models. A classification model was trained on the observed values (independent variables, see Table 1) based on the inputs being binary (e.g., sex and diarrhea) or multiclass (e.g., age and BMI), and the model predicted the outputs (dependent variable) of the binary class. 

Of the 24 independent variables used in this research, age and BMI were converted from continuous to categorical variables. Age was categorized based on age matrices [35], where ‘younger adults’ ranged between 20 and 34, ‘middle adults’ ranged between 35 and 64, and ‘older adults’ ranged between 65 and 90 years. Similarly, BMI was categorized based on the BMI metrics [36], with ‘underweight’ defined as below 18.50, ‘normal weight’ between 18.5 and 24.9, ‘overweight’ between 25 and 29.9, and ‘obese’ defined as 30 or greater (see Table 1). 

In addition, an improved alternative for dummy coding demonstrated in Table 1 was adapted from our previous study on the same dataset [31] in place of ‘one-hot-encoding’ [37]. This approach harnessed the capabilities and efficiency of AI modeling while leveraging interpretability and domain knowledge. Thus, the dummy coding facilitated the effective comprehension of the feature analysis. It is important to acknowledge that the model’s optimal performance may be affected due to biases introduced in this approach. 

However, it simultaneously equips healthcare providers with a well-defined understanding of the health status based on the features analyzed. For example, this research delved into inquiries, such as which age group or presence of diabetes had a more pronounced impact, whether patients taking medication for hypertension (ARBs and ACEIs) yielded benefits, whether individuals with diarrhea exhibited a higher predictability of requiring ICU admission, or if the combination of two variables (interaction effect) exerted a more substantial influence than a single variable.

### 2.4. Correlation Check

Tetrachoric correlation analysis was employed to acquire a more in-depth understanding of the effectiveness and suitability of 24 independent variables. We chose the tetrachoric correlation due to the categorical nature of our variables, which cannot be analyzed using the Pearson correlation analysis. In addition, the tetrachoric correlation can also handle skewness and outliers in the dataset, capture non-linear correlations between variables, and deal with ordinal data [38]. 

The analysis revealed that the correlation coefficients among all pairs of variables were generally low (<0.50), with one exception being the positive correlation observed between CKD stage 5 and the dependence on renal dialysis, presenting a correlation coefficient of 0.66. The correlation analysis was conducted solely for exploratory data analysis and was not used as a guiding principle for feature selection. The model’s accuracy can potentially be enhanced when two correlated features are part of the same dataset, as discussed by Deb et al. [39].

### 2.5. Data Splitting

The Scikit-learn library [40] randomly split the dataset into two: training (*N* = 4296) and test (*N* = 1075). The datasets (train and test) contained 8%, 11%, and 19% of the data from ‘MV’, ‘ICU’, and ‘IMCU’, respectively; similarly, 92%, 89%, and 81% of the data were from the ‘no MV’, ‘no ICU’, and ‘no IMCU’ classes, respectively.

### 2.6. Resampling Data

The data were found to be imbalanced based on the information above. Oversampling was utilized to balance uneven datasets. Synthesizing new examples instead of duplicating was performed using the **S**ynthetic **M**inority **O**ver-sampling **T**echniqu**E** (SMOTE) to balance the data [41]. SMOTE was applied to the training dataset, but the test dataset did not undergo any modifications to prevent data leakage issues [39].

## 3. Results

### 3.1. Cohort Description

The table below describes the categorical variables and corresponding dummy coding values.

We present the rest of the results in the following order: MV requirement analysis is reported first, followed by ICU admission and IMCU admission. Section 3 includes statistical analysis, model prediction, feature interpretability, and interactions. 

### 3.2. Statistical Analysis

Three individual chi-squares were used to estimate the predictive value of 24 independent variables in identifying individuals likely to require MV, or be admitted to ICU or IMCU [42].

As shown in Table 2, 12 of the 24 variables were statistically significant in predicting the likelihood of requiring MV. These variables included age, sex, diabetes, hypertension, CKD stages 1–4, CKD stage 5, heart failure, coronary artery disease, liver disease, pneumonia, diarrhea, and dependence on renal dialysis. The highest risk factors associated with MV were diarrhea (OR = 7.2), CKD stages 1–4 (OR = 2.89), and hypertension (OR = 2.75).

As shown in Table 3, among the 24 variables, 15 were statistically significant in predicting the likelihood of admitting to the ICU. These variables included age, BMI, sex, race, diabetes, hypertension, **C**hronic **O**bstructive **P**ulmonary **D**isease (COPD), CKD stages 1–4, CKD stage 5, heart failure, coronary artery disease, liver disease, pneumonia, diarrhea, and dependence on renal dialysis. The variables associated with the highest risk factors were diarrhea (OR = 8.86) and age (OR = 3.43), with older adults being 3.43-times more likely to be admitted to the ICU than younger adults. 

As shown in Table 4, 15 of the 24 variables were statistically significant in predicting the likelihood of admitting to the IMCU. These variables included age, sex, smoking status, diabetes, hypertension, COPD, CKD stages 1–4, CKD stage 5, heart failure, cardiac arrhythmias, coronary artery disease, pneumonia, ARBs, ACEIs, and diarrhea. The highest risk factors were diarrhea (OR = 2.86) and age (OR = 2.74), with older adults being 2.74-times more likely to be admitted to the IMCU than younger adults. 

Using traditional statistical approaches, multiple options exist for selecting the best key features. One of the most popular approaches involves both forward and backward stepwise approaches. We chose the backward Wald stepwise binary logistic regression method due to its conservativeness and low likelihood of introducing false positives to the model [43,44,45]. 

Several problems have been noted with this traditional approach. The biggest issue is that these approaches can overfit the model specifically for that sample, and therefore, selecting the key features can vary from sample to sample [46,47]. To compensate for this potential problem, a 10-fold cross-validation approach was used with Wald’s backward binary logistic regression. This validation routine randomly parsed the data into 9 separate training datasets of 573 patients and a 10th testing dataset with 574 patients. **M**ean **S**quared **E**rrors (MSEs) and R^2^ were compared in each fold. Lastly, a final imputed dataset was constructed from the features identified in the training dataset and compared with the values in the test dataset to obtain the final variable selection and R^2^. Table 5, Table 6 and Table 7 report the three separated Wald’s backward binary logistic regression results for MV, ICU, and IMCU.

As seen in Table 5, of the initial 24 features used to predict the likelihood to require MV, the following 14 are retained by the model: age, BMI, sex, race, diabetes, hypertension, CKD stages 1–4, cardiac arrhythmias, cerebrovascular disease, pneumonia, ARBs, ACEIs, diarrhea, and dependence on renal dialysis. These features were statistically significant in predicting MV requirement and accounted for approximately 20% of the variability in the model [χ^2^(8) =373.96, *p* < 0.001, Nagelkerke R^2^ = 0.20] with a 92.5% overall correct classification rate. Of these retained variables, diarrhea had the largest odds ratio in the multivariate binary logistic regression model; those diagnosed with diarrhea were 6.31-times more likely to require MV than those who did not.

As seen in Table 6, of the initial 24 features used to predict patients admitted to the ICU, the following 15 are retained by the model: age, BMI, sex, race, diabetes, hypertension, asthma, CKD stages 1–4, heart failure, cardiac arrhythmias, cerebrovascular disease, pneumonia, ARBs, ACEIs, and diarrhea. These features were statistically significant in predicting ICU admissions and accounted for approximately 26% of the variability in the model [χ^2^(8) = 374.96, *p* < 0.001, Nagelkerke R^2^ = 0.26] with an 89.4% overall correct classification rate. Of these retained variables, diarrhea had the highest odds ratio in the multivariate binary logistic regression model; those diagnosed with diarrhea were 8.52-times more likely to be admitted to the ICU than those who did not.

Lastly, Table 7 presents the results for predicting the likelihood of being admitted to the IMCU; of the initial 24 features, the following 14 were retained by the model: age, BMI, sex, race, ethnicity, diabetes, hypertension, COPD, CKD stages 1–4, heart failure, cardiac arrhythmias, pneumonia, ACEIs, and diarrhea. These features were statistically significant in predicting IMCU admissions and accounted for approximately 11% of the variability in the model [χ^2^(15) = 374.96, *p* < 0.001, Nagelkerke R^2^ = 0.11] with an 81.5% overall correct classification rate. Of these retained variables, diarrhea had the largest odds ratio in the multivariate binary logistic regression model; those diagnosed with diarrhea were 2.56-times more likely to be admitted to IMCU than those who did not.

To further assess the potential of invariances across gender, age, and ethnicity, sets of backward binary logistic regressions were conducted and cross-validated for each group. Table 8, Table 9 and Table 10 report all the variables we identified as important for predicting MV requirement and ICU and IMCU admissions across groups. 

The classification accuracy for MV by sex ranged from 91.5% for males to 93.6% for females. There were more significant differences in model accuracy between the age categories, with older adults having the lowest accuracy (89.7%) compared to younger adults (97.3%) and ‘middle’ adults (94.1%). The accuracy for the non-Hispanic and Hispanic groups was approximately equal for MV, with accuracy values of 92.4% and 92.7%, respectively. 

The highest overall accuracy across all groups was observed for predicting ICU admissions. All groups reported an accuracy value above 90% for predicting ICU admission, except for ethnicity, approaching 90% accuracy (non-Hispanic = 89.6% and Hispanic = 89.8%). 

When assessing IMCU admissions, the overall accuracy decreased, ranging from a low of 76.4% for older adults to a high of 90.5% for younger adults. As can be seen, some variabilities across the features are important for predicting MV, ICU, and IMCU outcomes among the demographic groups of sex, age, and ethnicity. This suggests the necessity of retaining demographic variables in all future analyses.

### 3.3. Predictive Analysis

#### 3.3.1. Model Performance

The models’ performances on the imbalanced dataset were assessed with three metrics: precision, recall, F1-score (weighted), and AUC. Precision measures how many of the positive predictions made by the classifier are correct, i.e., ‘of all the predicted severe cases, how many are actually severe?’ Recall quantifies how well the classifier identifies all positive cases, i.e., ‘what proportion of actual severe cases did the model correctly predict as severe?’ 

**Figure 2:**
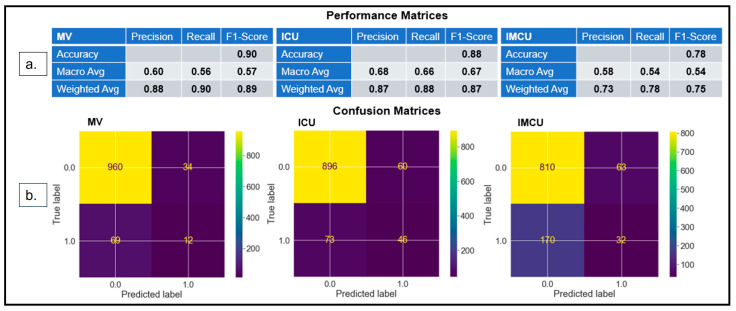
(**a**) *Performance matrices* show that the models’ performances in the test dataset yield an F1-score of 0.89 for MV, 0.87 for ICU admission, and 0.75 for IMCU. (**b**) *Confusion matrices* demonstrate that the models correctly classify 972 instances for MV, 942 instances for ICU, and 842 instances for IMCU.

F1 score balances precision and recall, considering both false positives and negatives. The harmonic mean of precision and recall is used to estimate the F1 score, where the best value is 1.0 and the worst value is 0.0 [48]. Upon training the RF classifier, we obtained F1-scores (Figure 2a) of 89% (precision: 88% and recall: 90%), 87% (precision: 87% and recall: 88%), and 75% (precision: 73% and recall: 78%), respectively, for MV, ICU, and IMCU. 

Model performance was relatively poor in predicting IMCU admissions because, in this case, the model displayed an inadequate performance in the majority class, which resulted in a poor F1 score. The above results are reported after the models have been optimized for hyperparameter tuning using the grid search K-fold cross-validation (10-fold) method [49,50]. 

Confusion matrices (Figure 2b) were used to assess the model’s classification, which indicated that the model accurately classified 972 (TP: 960 and TN: 12), 942 (TP: 896 and TN: 46), and 842 (TP: 810 and TN: 32) instances, and misclassified 103 (FP: 34 and FN: 69), 133 (FP: 60 and FN: 73), and 233 (FP: 63 and FN: 170) instances for MV, ICU, and IMCU, respectively. The model predicted that the IMCU had the lowest TP and highest FP, leading to low specificity and sensitivity.

The models’ performances were also evaluated using the AUC of the **R**eceiver **O**perating **C**haracteristic (ROC) analysis. A perfect model would provide an AUC of 1, and an uninformed model would provide an AUC of 0.5. A probability curve plots the TP versus the FP rate at different threshold values and distinguishes between ‘severity’ and ‘no-severity’ cases.

**Figure 3:**
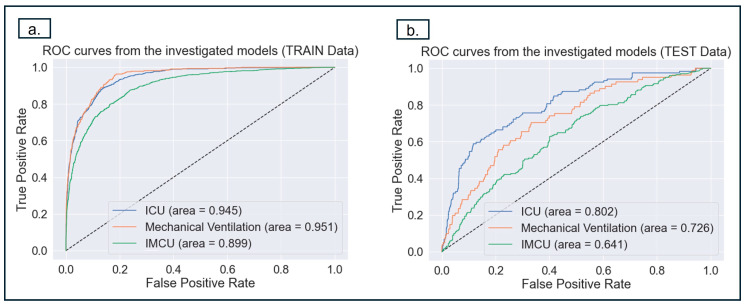
The figures above depict the *ROC analysis* to evaluate the models’ performances in predicting MV, ICU, and IMCU for training and test cohorts. (**a**) This shows the ROC curve for the training dataset (**b**). This shows ROC curve for the test data, the prediction of ICU achieves the best performance. It should be noted that FP is 1 minus the specificity (TN), which means that the closer FP is to 0, the higher the sensitivity (TN). Therefore, the point should be in the top-left corner of the ROC curve to obtain the optimal values for specificity and sensitivity.

The reported ROC-AUC (Figure 3a) values for the predictions of MV, ICU, and IMCU were 73% (95% CI: 0.66–0.79), 80% (95% CI: 0.75–0.85), and 64% (95% CI: 0.60–0.69), respectively. In the current study, AUC performance for MV and IMCU was poorer than ICU.

In the test data, we observed an inadequate model performance in predicting minority classes. The imbalanced nature of the dataset caused this discrepancy. The model is biased toward the data’s predominant nature to predict the majority class; for instance, predicting ‘ICU’ is lower in accuracy than ‘no ICU’.

#### 3.3.2. Model Interpretability

Three interpretability (post hoc) methods were used to explain the models’ performance. These included MDI (Mean Decrease in Impurity), Permutation Importance, and SHAP for being the most common interpretable techniques based on our background literature reviews [15,22,23,26,31]. 

**Figure 4:**
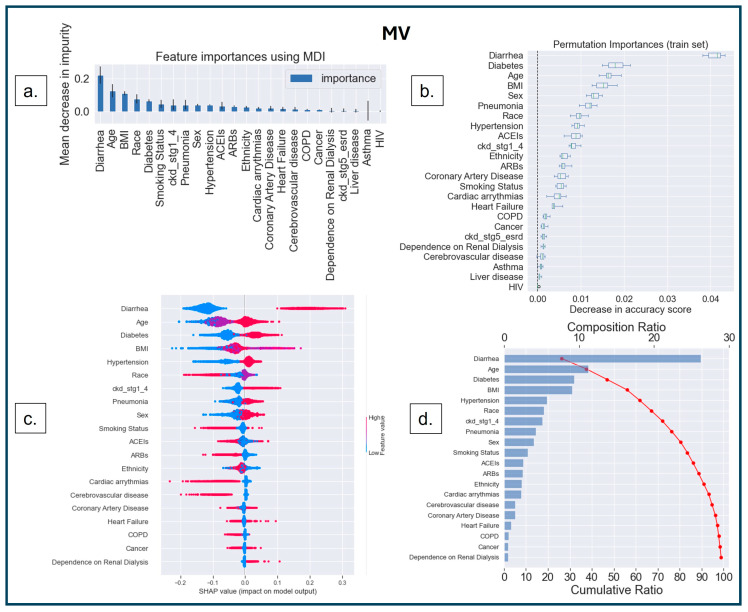
Feature interpretation. (**a**) Feature importance using MDI in MV; the most important features are depicted in the higher bars. (**b**) Features ranked (Y-axis) based on the decrease in accuracy (X-axis) under the effect of permutations. The ‘bee swarm plot’ (**c**) depicts the graphical representation of feature values based on dummy coding (Table 1), where red indicates higher values and blue indicates lower values. Each dot is representative of a patient and highlights which feature values correspond to SHAP values (X-axis). (**c**) The most important feature is diarrhea, followed by age, diabetes, BMI, and hypertension, the top 5 most important features in ranking order for predicting MV use. The ‘Waterfall plot’ (**d**) indicates that diarrhea results in a composition ratio of >25% (X-axis, top). Furthermore, the top 13 features accumulate 90% of the model’s cumulative ratio (X-axis, bottom).

**Figure 5:**
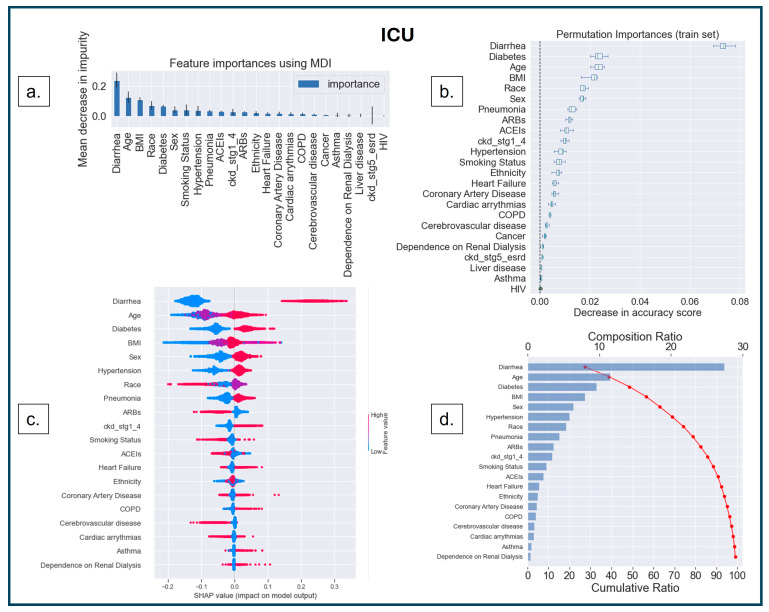
Feature interpretation. (**a**) Feature importance using MDI in ICU; the most important features are depicted in the higher bars. (**b**) Features ranked (Y-axis) based on the decrease in accuracy (X-axis) under the effect of permutations. The ‘bee swarm plot’ (**c**) shows that the most important feature is diarrhea, followed by age, diabetes, BMI, and sex, the top 5 most important features in ranking order for predicting ICU. The ‘Waterfall plot’ (**d**) indicates that diarrhea results in a composition ratio of >25% (X-axis, top). Furthermore, the top 12 features accumulate 90% of the model’s cumulative ratio (X-axis, bottom).

**Figure 6:**
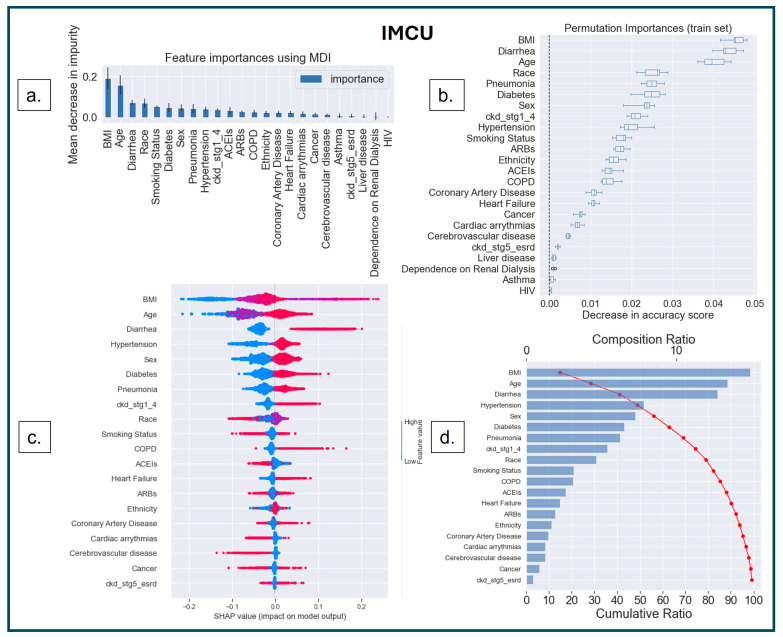
Feature interpretation. (**a**) Feature importance using MDI in IMCU; the most important features are depicted in the higher bars. (**b**) Features ranked (Y-axis) based on the decrease in accuracy (X-axis) under the effect of permutations. The ‘bee swarm plot’ (**c**) shows that the most important feature is BMI, followed by age, diarrhea, hypertension, and sex, the top 5 most important features in ranking order. The ‘Waterfall plot’ (**d**) indicates that BMI results in a composition ratio of ~20% (X-axis, top). Furthermore, the top 13 features accumulate 90% of the model’s cumulative ratio (X-axis, bottom).

The first interpretability method was MDI, based on the Gini impurity values of the RF, where splitting rules maximize impurity reduction [51,52,53,54]. Impurity indicates how well a node can classify a dataset. The Gini index determines the impurity by calculating the probability of misclassifying a randomly selected element from the dataset [55]. The term ‘impurity’ determines how homogeneous or mixed the classes are in a node, where ‘zero’ impurity has only one class, and a node with maximum impurity has an equal mix of all classes [52]. MDI measures how much each feature reduces the impurity of the nodes where it is used to split the data, and it calculates feature importance as the sum of impurity decreases across all the splits that include the feature, averaging over all the trees in the ensemble [52]. The higher the MDI (Figure 4a, Figure 5a and Figure 6a), the more influential the feature is for the model. A split with a significant decrease in impurity is important for the RF classifier; consequently, the feature and the corresponding split are essential for the model’s decision.

The second method was model-agnostic interpretability based on permutation-based analysis [56,57]. In this method, the values of an input feature on the dataset are shuffled, and the model’s change in prediction accuracy is recorded for the shuffled feature. The exact process is repeated for other features, keeping the rest of the dataset unshuffled. The features are then ranked based on the variability in model performance [56,57]. This permutation-based feature ranking (Figure 4b, Figure 5b and Figure 6b) provides the most important values for the model’s performance.

The third (another model agnostic) interpretability method is based on the cooperative game theory that computes SHAP values for each player in a multiplayer game to understand the outcome [58,59,60,61]. Shapley values are measured for each feature to identify its contribution to changing the model’s decision. The SHAP value for a particular feature is calculated as the change in the difference in prediction from randomly sampling the feature value from a distribution compared to the average prediction across all data instances (see Figure 4c,d, Figure 5c,d and Figure 6c,d). 

SHAP and MDI have a similar performance when identifying important features [51]; however, SHAP considers the local and global distributions of features, whereas MDI only provides global importance. SHAP overestimates irrelevant features over relevant features under binary networks [62] and incorrectly identifies important features in out-of-distribution scenarios [63]. However, the permutation-based approach is unique as it can capture other important features undetected by SHAP [64].

The ‘bee swarm plot’ (Figure 4c, Figure 5c and Figure 6c) depicts the SHAP values of each feature distributed for all populations, where the sub-categories of each feature are presented as a ‘heatmap’ as per the dummy coding (see Table 1). ‘Cool color’ (blue) represents a sub-category coded by a lower number (i.e., ‘0’ denotes ‘no-diabetes’), and ‘warm color’ (red) represents a higher number (i.e., ‘1’ denotes ‘diabetes’). On the X-axis, the ‘0’ in the middle of the SHAP scale indicates a ‘Neutral Point’—a value toward the right supports a positive decision (predicting ‘MV’, ‘ICU’, or ‘IMCU’); consequently, a value toward the left supports a negative decision (predicting ‘no-MV’, ‘no-ICU’, or ‘no-IMCU’). Features are arranged along the Y-axis based on their importance in ranking order; the more important features are placed at the top, which is assigned using their absolute Shapley values.

SHAP analysis can also be comprehended through the ‘waterfall plot’ (Figure 4d, Figure 5d and Figure 6d), where each feature’s contribution can be analyzed to understand model explainability. The features on the Y-axis are ranked based on the compositional score (X-axis, top) estimated as the mean of SHAP values across the population for the presented feature. The cumulative score (X-axis, bottom) shows the additive values of features on the model that explain the model’s interpretation. 

In Figure 4a, the top five features that determine a model’s prediction for MV include diarrhea, age, BMI, race, and diabetes, in ranking order, using MDI. The Permutation Importance (Figure 4b) indicates similar top five features; however, sex (ranked 5th) is more important than race (ranked 7th). The SHAP analysis (Figure 4c,d) agrees with the other two methods over the top four features: diarrhea, age, diabetes, and BMI. It ranked hypertension 5th among the most important features. Despite the slight difference, all interpretability methods tend to agree on the most important features contributing to the model’s decision making.

Figure 4d shows that the top 13 features contribute 90% to the model’s interpretation. Among them, five are from sociodemographic characteristics (age, race, sex, smoking status, and ethnicity), six are from comorbidities (diarrhea, diabetes, BMI, hypertension, pneumonia, and CKD stages 1–4), and two are from the medication category (ACEIs and ARBs). Thus, the findings suggest that the trained models can use the information from each feature category to make a successful prediction. 

The features were analyzed similarly for ICU, where MDI (Figure 5a), Permutation Importance (Figure 5b), and SHAP (Figure 5c,d) show that there is consistency across the feature importance for both models: ICU and MV. SHAP showed that sex was among the top 5 features instead of race, as indicated by MDI and Permutation Importance. Figure 5d indicates that the top 12 features contributed to the model’s 90% interpretation. Among them, four were from sociodemographic characteristics (age, sex, race, and smoking status), six were from comorbidities (diarrhea, diabetes, BMI, hypertension, pneumonia, and CKD stages 1–4), and two were from the medication category (ARBs, and ACEIs). We found one less socio-demographic feature (ethnicity) than in MV, contributing to the 90% decision.

In the IMCU, MDI (Figure 6a) and Permutation Importance (Figure 6b) demonstrated that BMI, age, diarrhea, and race are the top 4 most important features. However, SHAP (Figure 6c,d) indicated that the top 3 features are identical to the other two methods. In SHAP, BMI is the most important feature, followed by age, diarrhea, hypertension, and sex.

Figure 6d shows that the top 13 features contribute to the model’s 90% interpretation. Among them, four were from sociodemographic characteristics (age, sex, race, and smoking status), eight were from comorbidities (BMI, diarrhea, hypertension, diabetes, pneumonia, CKD stages 1–4, COPD, and heart failure), and one was from the medication category (ACEIs). Compared to MV, we found that one sociodemographic (ethnicity) and one medication (ARBs) feature were replaced by COPD and heart failure. Similarly, COPD and heart failure were more important features than ARBs compared to ICU, contributing to the 90% interpretation.

It is worth noting that the results show similar features between MV and ICU, compared to IMCU, as patients requiring MV were also admitted to the ICU.

#### 3.3.3. SHAP Dependence Plot

A ‘SHAP dependence plot’ demonstrates how the model output differs depending on the interaction of two features [16,65]. By examining the scatterplot, we can analyze the pattern and trend of the relationship between a variable and the model’s output while considering the other variable [66,67]. If there is an interaction effect, it will be evident through distinct patterns along the Y-axis [16,66]. 

**Figure 7:**
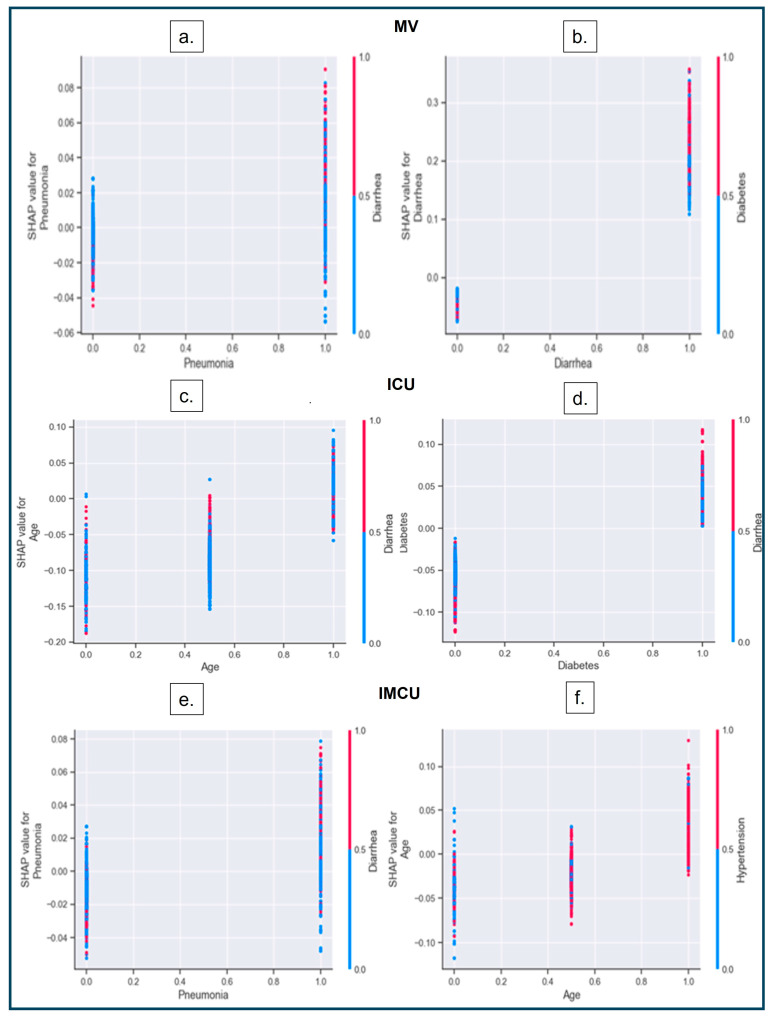
SHAP dependence plot. (**a**,**b**) Interactions between pneumonia–diarrhea and diarrhea–diabetes have a higher impact on predicting MV. An increase in the interaction between diarrhea (red dots) and pneumonia (scattered dots on the right) impacts the model’s output (higher SHAP values). (**a**) Higher density of red dots in the presence of pneumonia (indicated as categorical values on the x−axis), resulting in an increased likelihood of MV (shown as greater SHAP values) during the presence of two variables. (**c**,**d**) Interactions between age–diarrhea and diabetes–diarrhea have a higher impact on predicting ICU admissions. In (**c**), younger and middle-aged adults, diarrhea shows a sharp rise in prediction (higher SHAP values with red dots); however, diarrhea does not affect the older population much (less density of red dots). The X-axis of (**c**) represents the three different age categories (‘younger, ‘middle’, and ‘older’ adults). (**e**,**f**) Interactions between pneumonia–diarrhea and age–hypertension have higher impacts on predicting IMCU admissions.

Figure 7 below shows that the sub-groups of one of the categorical variables are on the X-axis, whereas the sub-groups of other categorical variables are on the Y-axis (right). The Y-axis (left) represents the SHAP value that interacts between the two categorical variables associated with each patient, represented as dots.

## 4. Discussion

The success of Random Forest classifiers in previous studies [23,24,27,30,31,32] led us to select the same approach for our current study, and the performance of our models after training is comparable with previous studies. This suggests that ML can be a valuable tool for predicting the severity of COVID-19 disease for rapid therapeutic interventions, like MV requirement, ICU, or IMCU admission. Under high-demand conditions, healthcare systems can rely on an alternate, fast intelligence system for therapeutic decision making under critical care. Our study contributes toward understanding COVID-19 disease severity in a large sample from South Florida and identifies essential features utilizing interpretability approaches in AI/ML techniques. This study identified the primary aspects of eHR data that play a crucial role in COVID-19 disease severity. 

We extended the feature analysis by comparing multiple interpretability techniques. Our analysis explored how each feature impacts the critical condition and how the interplay between features contributes to the severity of COVID-19 disease.

In this study, we trained three classifiers (RF) to predict each of the three different conditions: (1) MV requirement, (2) ICU, and (3) IMCU admission. We trained the models using 24 independent variables containing information on patients’ sociodemographic characteristics, comorbidities, and medications. The F1-scores (weighted) across our three prediction models were 0.89 for MV, 0.87 for ICU, and 0.75 for IMCU, with an AUC of 0.73 for MV, 0.80 for ICU, and 0.64 for IMCU. This performance was comparable to previous findings [15,19,21,25]. It is worth noting that the model’s accuracy was higher for MV and ICU compared to IMCU. 

There is evidence of a type I error in predicting IMCU, possibly due to misclassifying patients’ most important features (rank-wise) as false positives, which may not be true in actual cases and are better predictors for MV or ICU instead. In addition, IMCU patients may comprise two different populations: one directly admitted to the IMCU during their initial hospitalization, and another group stepped down from initial hospitalization to the ICU. Thus, the patients in the IMCU have a large diversity of risk factors, and it might be hard for the classifier to learn a better decision boundary. Also, there is evidence of type II errors in predicting IMCU admissions from ‘other COVID-19 patients’ who have not experienced severe events (MV, ICU, and IMCU). In those cases, presumably, the feature distribution of the ‘other COVID-19 patients’ class (‘no-MV’, ‘no-ICU’, and ‘no-IMCU’) may not be distinct from IMCU as the illness of the patient admitted to the IMCU is less severe compared to those in the MV or ICU groups. Thus, the model failed to distinguish features between the two classes (IMCU vs. ‘no-MV’, ‘no-ICU’, and ‘no-IMCU’, when taken together), resulting in more false negatives.

We utilized SHAP analysis to better understand the model’s decision and compared it with other interpretability methods: MDI and Permutation Importance. The SHAP analysis showed that the top five features (diarrhea, age, diabetes, BMI, and hypertension) for predicting MV were similar across other interpretive methods but in different ranking orders (MDI: diarrhea, age, BMI, race, diabetes; Permutation Importance: diarrhea, diabetes, age, BMI, and sex).

In the ICU, the SHAP analysis showed the top five features (diarrhea, age, diabetes, BMI, and sex), which were almost consistent with the other two interpretability methods (MDI: diarrhea, age, BMI, race, and diabetes; Permutation Importance: diarrhea, diabetes, age, BMI, and race). This shows that the other two methods placed race in the top five features instead of sex.

Similarly, in the IMCU, the SHAP analysis showed the top five features (BMI, age, diarrhea, hypertension, and sex) that are only matched for the top three features with the other two interpretability methods (MDI: BMI, age, diarrhea, race, and smoking status; Permutation Importance: BMI, diarrhea, age, race, and pneumonia). As discussed, IMCU is less severe than the other two disease severities (MV and ICU); hence, the model interprets the features differently than the other two disease severities. These findings were consistent with previous research on the clinical attributes and the prevalence of coexisting medical conditions in COVID-19 patients [15,17,20,25]. Even though limited studies compare feature importance across severe conditions, it is important to highlight that only one such study compared feature importance across different age groups, and the risk factors associated with COVID-19 vary across ages [68].

This research investigated how patients with compromised health conditions contribute to worsening the severity of COVID-19 disease. These include diarrhea, diabetes, hypertension, pneumonia, and CKD stages 1–4. This research revealed that patients on medications such as ARBs and ACEs, commonly used to manage high blood pressure and heart failure, decreased the likelihood of COVID-19 disease severity. Other researchers have also reported the protective nature of the medications (ARBs and ACEs) for hypertension [69,70,71,72] and agree with our findings. 

Finally, we also found some crucial interactions across features. Patients with pneumonia and diabetes, as well as diabetes and diarrhea, are more likely to require MV. In the ICU, diarrhea affects middle-aged adults the most and interacts strongly with diabetes. In the IMCU, the interaction between diarrhea and pneumonia, as well as hypertension and middle-aged or older adults, increase the risk of severity.

We found some common features for those reported as significant in the chi-squared test and features retained by binary logistic regression models, reported in the statistical results, with the top features reported in the three interpretability approaches. The common features reported in MV include age, sex, diarrhea, diabetes, hypertension, CKD stages 1–4, and pneumonia. Race and BMI stand out as top features across interpretability approaches, but they were not significant in the chi-squared test. 

In the ICU, age, sex, race, BMI, diarrhea, diabetes, hypertension, and pneumonia were important features in all statistical (chi-square and binary logistic regression) and interpretability methods. Lastly, in the IMCU, age, sex, diarrhea, diabetes, hypertension, CKD stages 1–4, and pneumonia were important features. BMI, race, and smoking status were important across all three interpretability approaches, but not in the statistical methods.

It is important to note that heart-related comorbidities significant in the statistical methods for all three severity conditions were not among the top 10 important features across the three interpretability techniques. 

Not many studies have analyzed features using traditional statistics and ML interpretable techniques, as explored in the current study. Wu et al. [73] used seven different interpretability techniques to identify important features from lab biomarker data for predicting COVID-19 disease severity. However, the several biomarkers analyzed in this study are not commonly administered in regular checkups nor frequently tested by clinicians. Thus, our study uniquely contributes to identifying important features over easily accessible eHR data across different COVID-19 severities of disease.

## 5. Limitations

We had no opportunity to intervene in the clinicians’ data collection process in the current study. Re-designing the prospective study may help eliminate ‘patient selection’, ‘patient interaction’, and ‘clinician reporting’ biases [74]. Data were collected when there was a surge of in-patients along with a high rate of death and severity of illness due to limited access to COVID-19 treatments (medications and vaccinations), leading to instances of incomplete, missing, or inaccurate clinical reporting. 

Some of these variables had to be removed from the dataset due to unavailability for many patients, such as ‘sputum at admission’ and ‘fever’. This information could have improved our models’ predictions if reported properly. Furthermore, the sociodemographic distribution of COVID-19 patients in South Florida could influence the model’s performance [74,75,76], leading to a better performance in certain sub-groups (e.g., sex, age, and ethnicity) with a greater population density.

Similarly, the imbalanced test dataset posed a challenge for the model in predicting the minority class (severe cases) accurately. It is important to reduce type II errors where the model fails to detect the minority class, as those are crucial for patient recovery from the severity of disease. Thus, clinicians should not solely rely on the model’s predictions, but instead use current clinical trends and judgments. 

Models can still perform poorly under outliers and unseen cases due to biased estimations and learning about spuriously correlated features, despite the great strides made to improve the models’ performance [74,75,76,77].

Thus, to aid clinicians in stratifying the most important features, we kept all independent variables and avoided feature selection based on statistical analysis, which could lead to underperformance. 

## 6. Future Direction

In our future work, we will explore the case-specific underperformance of our models, as we found that IMCU prediction results in lower accuracy compared to the other two models. Data augmentation and feature engineering [10,75] based on the significant features reported in the statistics can also help optimize the model’s performance. Specifically, feature selection methods, such as **U**niform **M**anifold **A**pproximation and **P**rojection (UMAP), have been shown to significantly improve a classifier’s performance when predicting COVID-19 severity [78]. Hence, we will implement UMAP feature selection in our future works for a more robust performance.

Second, we will explore a multiclass classifier that simultaneously predicts all three severity cases rather than training separate models for each condition. A comprehensive understanding of disease progression can enhance the transparent and trustworthy knowledge of end users and aid them in making better-informed decisions with the model.

In addition to multiclass classifications, we will utilize SHAP scores to cluster different patient groups, as Khadem et al. [79] reported, identifying the most susceptible cluster based on mortality counts. Cluster analysis may be necessary in future studies to identify the clusters representing a higher severity risk of COVID-19.

## 7. Conclusions

Developing an AI-driven decision support system for predicting the critical clinical events of in-patients with COVID-19 disease not only addresses the immediate needs of the pandemic, but also advances the field of AI/ML in healthcare. By leveraging cutting-edge technologies and algorithms, such as ML, researchers and healthcare professionals can unlock the potential of data-driven insights to transform patient care. The application of AI/ML in healthcare extends beyond the COVID-19 disease, holding promise for improving diagnosis, treatment selection, disease surveillance, and patient outcomes across various medical specialties and healthcare settings. By pushing the boundaries of AI/ML in healthcare, we can foster innovation, enhance decision making, and ultimately improve health outcomes for individuals and populations. This knowledge empowers public health authorities to proactively plan and implement targeted interventions, mitigating the impact of disease outbreaks and optimizing healthcare delivery.

## Figures and Tables

**Table 1:** Parameters and characteristics.

Dummy Coding		
**Patients’ Characteristics**	Age	‘Young adults’: 0, ‘Middle adults’: 1, ‘Older adults’: 2
	Sex	‘Female’: 0, ‘Male’: 1
	Race	‘Black’: 0, ‘Others’: 1, ‘White’: 2
	Ethnicity	‘Hispanic’: 0, ‘Not Hispanic’: 1
	Smoking Status	‘Never’: 0, ‘Former’: 1,‘Current’: 2
**Pre-hospital Comorbidities**	COPD	‘False’: 0, ‘True’: 1
	Kidney Disease (stg1_4)	‘False’: 0, ‘True’: 1
	Kidney Disease (stg5)	‘False’: 0, ‘True’: 1
	Diarrhea	‘No Diarrhea’: 0, ‘Diarrhea’: 1
	Hypertension	‘No Hypertension’: 0, ‘Hypertension‘: 1
	Diabetes	‘No Diabetes’: 0, ‘Diabetes‘: 1
	Pneumonia	‘No Pneumonia‘: 0, ‘Pneumonia‘: 1
	Heart Failure	‘False’: 0, ‘True’: 1
	Cardiac Arrhythmias	‘False’: 0, ‘True’: 1
	Coronary Artery Disease	‘False’: 0, ‘True’: 1
	Dependence on Renal Dialysis	‘No’: 0, ‘Yes’: 1
	Cerebrovascular Disease	‘No’: 0, ‘Yes’: 1
	BMI	‘Underweight’: 0, ‘Normal Weight’: 1, ‘Overweight’: 2, ‘Obesity’: 3
	Liver Disease	‘No’: 0, ‘Yes’: 1
	Asthma	‘No’: 0, ‘Yes’: 1
	HIV	‘No’: 0, ‘Yes’: 1
	Cancer	‘No’: 0, ‘Yes’: 1
**Medications**	ARBs	‘No’: 0, ‘Yes’: 1
	ACEIs	‘No’: 0, ‘Yes’: 1

Dummy coding is adopted from our previous study [31].

**Table 2:** Significant features across patients’ likelihood to require MV.

Features	No MV (*N* = 4964)		MV (*N* = 407)					
	*N*	%	*N*	%	χ^2^	df	*p*	OR
**Age**								
**Young adult**	609	12.3	22	5.4	51.43	2	<0.001	2.74
**Middle Adult**	2353	47.4	150	36.9				
**Older Adult**	2002	40.3	235	57.7				
**BMI**								
**Underweight**	67	1.3	8	2.0	4.98	3	0.173	1.77
**Normal**	821	16.5	54	13.3				
**Overweight**	1703	34.3	134	32.9				
**Obese**	2373	47.8	211	51.8				
**Sex (Male)**	2478	49.9	237	58.2	10.40	1	0.001	1.4
**Race**								
**Black**	1553	31.3	123	30.2	5.58	2	0.061	1.09
**Other**	2540	51.2	229	56.3				
**White**	871	17.5	55	13.5				
**Ethnicity (Not Hispanic)**	3317	66.8	262	64.4	1.01	1	0.314	0.9
**Smoking Status**								
**Never**	4121	83.0	330	81.1	1.01	2	0.603	1.29
**Former**	716	14.4	65	16.0				
**Current**	127	2.6	12	2.9				
**Diabetes**	1934	39.0	240	59.0	62.50	1	<0.001	2.25
**Hypertension**	3218	64.8	340	83.5	58.90	1	<0.001	2.75
**COPD**	425	8.6	46	11.3	3.53	1	0.060	1.36
**Asthma**	151	3.0	14	3.4	0.20	1	0.655	1.135
**CKD Stages 1 to 4**	744	15.0	117	28.7	52.90	1	<0.001	2.89
**CKD Stage 5**	156	3.1	25	6.1	10.40	1	0.001	2.02
**Heart Failure**	663	13.4	80	19.7	12.52	1	<0.001	1.59
**Cancer**	284	5.7	29	7.1	1.35	1	0.245	1.26
**Cardiac Arrhythmias**	626	12.6	48	11.8	0.23	1	0.632	0.93
**Cerebrovascular Disease**	318	6.4	19	4.7	1.93	1	0.165	0.72
**Coronary Artery Disease**	770	15.5	90	22.1	12.19	1	<0.001	1.55
**Liver Disease**	102	2.1	16	3.9	6.16	1	0.013	1.95
**HIV**	53	1.1	3	0.7	0.40	1	0.528	0.69
**Pneumonia**	1992	40.1	214	52.6	24.09	1	<0.001	1.65
**ARBs**	1283	25.8	110	27.0	0.27	1	0.601	1.06
**ACEIs**	1710	34.4	150	36.9	0.96	1	0.327	1.11
**Diarrhea**	613	12.3	205	50.4	421.16	1	<0.001	7.2
**Dependence on Renal Dialysis**	4846	97.6	382	93.9	20.58	1	<0.001	2.69

Individual chi-square results of the 24 demographic features predicting MV requirement.

**Table 3:** Significant features for patients’ likelihood of being admitted to the ICU.

Features	No ICU (*N* = 4775)		ICU (*N* = 596)					
	*N*	%	*N*	%	χ^2^	df	*p*	OR
**Age**								
**Young adult**	600	95.1	31	4.9	70.19	2	<0.001	3.43
**Middle Adult**	2275	90.9	228	9.1	-	-	-	-
**Older Adult**	1900	84.9	337	15.1	-	-	-	-
**BMI**				0.0				
**Underweight**	66	88	9	12.0	10.51	3	0.015	0.667
**Normal**	801	91.5	74	8.5	-	-	-	-
**Overweight**	1642	89.4	195	10.6	-	-	-	-
**Obese**	2266	87.7	318	12.3	-	-	-	-
**Sex (Male)**	2368	87.2	347	12.8	15.79	1	<0.001	1.42
**Race**				0.0				
**Black**	1512	90.2	164	9.8	16.76	2	<0.001	0.86
**Other**	2416	87.3	353	12.7	-	-	-	-
**White**	847	91.5	79	8.5	-	-	-	-
**Ethnicity (Not Hispanic)**	1578	88.1	214	11.9	1.95	1	0.163	0.881
**Smoking Status**				0.0				
**Never**	3969	89.2	482	10.8	1.94	2	0.379	1.23
**Former**	685	87.7	96	12.3	-	-	-	-
**Current**	121	87.1	18	12.9	-	-	-	-
**Diabetes**	1818	83.6	356	16.4	103.16	1	<0.001	2.41
**Hypertension**	3066	86.2	492	13.8	79.71	1	<0.001	2.64
**COPD**	395	83.9	76	16.1	13.29	1	<0.001	1.62
**Asthma**	140	84.8	25	15.2	2.84	1	0.092	1.45
**CKD Stages 1 to 4**	699	81.2	162	18.8	61.92	1	<0.001	2.78
**CKD Stage 5**	150	82.9	31	17.1	6.91	1	0.009	1.69
**Heart Failure**	623	83.8	120	16.2	22.33	1	<0.001	1.68
**Cancer**	271	86.6	42	13.4	1.82	1	0.178	1.26
**Cardiac Arrhythmias**	599	88.9	75	11.1	0.00	1	0.978	1
**Cerebrovascular Disease**	307	91.1	30	8.9	1.75	1	0.185	1.23
**Coronary Artery Disease**	732	85.1	128	14.9	14.89	1	<0.001	1.51
**Liver Disease**	97	82.2	21	17.8	5.49	1	0.019	1.76
**HIV**	53	94.6	3	5.4	1.89	1	0.169	1.51
**Pneumonia**	1907	86.4	299	13.6	22.91	1	<0.001	1.51
**ARBs**	1232	88.4	161	11.6	0.41	1	0.524	1.06
**ACEIs**	1637	88	223	12.0	2.30	1	0.130	1.15
**Diarrhea**	511	62.5	307	37.5	683.48	1	<0.001	8.86
**Dependence on Renal Dialysis**	111	77.6	32	22.4	18.95	1	<0.001	2.38

Individual chi-square results of the 24 demographic features predicting ICU admission.

**Table 4:** Significant features for patients’ likelihood of being admitted to IMCU.

Features	No IMCU (*N* = 4361)		IMCU (*N* = 1010)					
	*N*	%	*N*	%	χ^2^	df	*p*	OR
**Age**								
**Young adult**	566	89.7	65	10.3	78.79	2	<0.001	2.74
**Middle Adult**	2094	83.7	409	16.3	-	-	-	-
**Older Adult**	1701	76.0	536	24.0	-	-	-	-
**BMI**				0.0				
**Underweight**	66	88.0	9	12.0	5.79	3	0.122	1.78
**Normal**	729	83.3	146	16.7	-	-	-	-
**Overweight**	1486	80.9	351	19.1	-	-	-	-
**Obese**	2080	80.5	504	19.5	-	-	-	-
**Sex (Male)**	2221	83.6	435	16.4	20.27	1	<0.001	1.37
**Race**				0.0				
**Black**	1372	81.9	304	18.1	1.30	2	0.522	1.09
**Other**	2232	80.6	537	19.4	-	-	-	-
**White**	757	81.7	169	18.3	-	-	-	-
**Ethnicity (Not Hispanic)**	1458	81.4	334	18.6	0.05	1	0.825	1.02
**Smoking Status**				0.0				
**Never**	3641	81.8	810	18.2	6.84	2	0.033	1.28
**Former**	608	77.8	173	22.2	-	-	-	-
**Current**	112	80.6	27	19.4	-	-	-	-
**Diabetes**	1648	75.8	526	24.2	69.50	1	<0.001	1.79
**Hypertension**	2748	77.2	810	22.8	108.31	1	<0.001	2.38
**COPD**	331	70.3	140	29.7	40.32	1	<0.001	1.96
**Asthma**	129	78.2	36	21.8	1.01	1	0.314	1.21
**CKD Stages 1 to 4**	622	72.2	239	27.8	53.84	1	<0.001	1.86
**CKD Stage 5**	130	71.8	51	28.2	10.78	1	0.001	1.73
**Heart Failure**	540	72.7	203	27.3	40.97	1	<0.001	1.78
**Cancer**	242	77.3	71	22.7	3.28	1	0.070	1.29
**Cardiac Arrhythmias**	527	78.2	147	21.8	4.56	1	0.033	1.24
**Cerebrovascular Disease**	272	80.7	65	19.3	0.06	1	0.815	1.03
**Coronary Artery Disease**	642	74.7	218	25.3	28.72	1	<0.001	1.59
**Liver Disease**	92	78.0	26	22.0	0.82	1	0.364	1.23
**HIV**	48	85.7	8	14.3	0.76	1	0.384	0.72
**Pneumonia**	1706	77.3	500	22.7	36.55	1	<0.001	1.5
**ARBs**	1076	77.2	317	22.8	19.24	1	<0.001	1.4
**ACEIs**	1480	79.6	380	20.4	4.92	1	0.026	1.17
**Diarrhea**	531	64.9	287	35.1	167.52	1	<0.001	2.86
**Dependence on Renal Dialysis**	108	75.5	35	24.5	3.09	1	0.079	1.14

Individual chi-square results of the 24 demographic features predicting IMCU admission.

**Table 5:** Backward binary logistic regression for predicting MV requirement.

							95% CI for OR	
Features	B	SE	Wald	df	*p*	OR	Lower	Upper
**Age**	0.43	0.11	15.27	1	<0.001	1.54	1.24	1.91
**BMI**	0.14	0.08	3.31	1	0.069	1.15	0.99	1.34
**Sex**	0.32	0.11	7.94	1	0.005	1.38	1.10	1.72
**Black**			9.48	2	0.009			
**Other**	0.12	0.13	0.81	1	0.369	1.12	0.87	1.44
**White**	−0.41	0.19	4.81	1	0.028	0.67	0.46	0.96
**Diabetes**	0.49	0.12	16.71	1	<0.001	1.63	1.29	2.05
**Hypertension**	0.73	0.17	18.98	1	<0.001	2.08	1.50	2.89
**CKD Stages 1 to 4**	0.57	0.14	17.47	1	<0.001	1.76	1.35	2.30
**Cardiac Arrhythmias**	−0.38	0.18	4.34	1	0.037	0.69	0.48	0.98
**Cerebrovascular Disease**	−0.52	0.26	3.90	1	0.048	0.60	0.36	1.00
**Pneumonia**	0.35	0.11	10.24	1	0.001	1.43	1.15	1.77
**ARBs**	−0.44	0.13	11.11	1	<0.001	0.65	0.50	0.84
**ACEIs**	−0.28	0.12	4.97	1	0.026	0.76	0.60	0.97
**Diarrhea**	1.84	0.11	268.20	1	<0.001	6.31	5.06	7.87
**Dependence on Renal Dialysis**	0.69	0.26	7.01	1	0.008	1.99	1.20	3.30
**Constant**	−4.94	0.30	267.94	1	<0.001	0.01		

**Note.** Nagelkerke R square = 0.202; classification = 92.5%.

**Table 6:** Backward binary logistic regression for predicting ICU admission.

							95% CI for OR	
Features	B	SE	Wald	df	*p*	OR	Lower	Upper
**Age**	0.46	0.10	22.70	1	<0.001	1.58	1.31	1.91
**BMI**	0.22	0.07	10.66	1	0.001	1.25	1.09	1.42
**Sex**	0.41	0.10	16.89	1	<0.001	1.51	1.24	1.83
**Black**			23.87	2	<0.001			
**Other**	0.32	0.11	7.97	1	0.005	1.38	1.10	1.72
**White**	−0.36	0.16	4.78	1	0.029	0.70	0.51	0.96
**Diabetes**	0.61	0.10	34.67	1	<0.001	1.84	1.50	2.26
**Hypertension**	0.72	0.14	24.92	1	<0.001	2.04	1.54	2.71
**Asthma**	0.57	0.25	5.08	1	0.024	1.76	1.08	2.88
**CKD Stages 1 to 4**	0.43	0.12	12.43	1	<0.001	1.54	1.21	1.95
**Heart Failure**	0.41	0.14	8.63	1	0.003	1.51	1.15	1.99
**Cardiac Arrhythmias**	−0.37	0.16	5.27	1	0.022	0.69	0.50	0.95
**Cerebrovascular Disease**	−0.44	0.22	3.93	1	0.047	0.65	0.42	1.00
**Pneumonia**	0.23	0.10	5.57	1	0.018	1.26	1.04	1.52
**ARBs**	−0.51	0.12	19.55	1	<0.001	0.60	0.48	0.75
**ACEIs**	−0.29	0.11	7.20	1	0.007	0.75	0.60	0.92
**Diarrhea**	2.14	0.10	463.52	1	<0.001	8.52	7.01	10.36
**Constant**	−4.98	0.27	346.66	1	<0.001	0.01		

**Note.** Nagelkerke R square = 0.26; classification = 89.4%.

**Table 7:** Backward binary logistic regression for predicting IMCU admission.

							95% CI for OR	
Features	B	SE	Wald	df	*p*	OR	Lower	Upper
**Age**	0.27	0.07	14.12	1	<0.001	1.30	1.14	1.50
**BMI**	0.15	0.05	9.18	1	0.002	1.16	1.06	1.28
**Sex**	0.32	0.07	18.76	1	<0.001	1.38	1.19	1.59
**Black**			7.04	2	0.030			
**Other**	0.22	0.11	4.22	1	0.040	1.24	1.01	1.53
**White**	−0.08	0.12	0.51	1	0.476	0.92	0.74	1.15
**Ethnicity**	0.17	0.10	2.88	1	0.090	1.19	0.97	1.45
**Diabetes**	0.27	0.08	11.75	1	<0.001	1.31	1.12	1.53
**Hypertension**	0.59	0.10	32.14	1	<0.001	1.80	1.47	2.20
**COPD**	0.35	0.12	8.66	1	0.003	1.42	1.12	1.78
**CKD Stage 1 to 4**	0.22	0.10	5.17	1	0.023	1.25	1.03	1.51
**Heart Failure**	0.29	0.11	7.07	1	0.008	1.34	1.08	1.66
**Cardiac Arrhythmias**	−0.21	0.12	3.10	1	0.078	0.81	0.64	1.02
**Pneumonia**	0.29	0.07	15.54	1	<0.001	1.34	1.16	1.54
**ACEIs**	−0.24	0.08	8.40	1	0.004	0.79	0.67	0.93
**Diarrhea**	0.94	0.09	119.11	1	<0.001	2.56	2.17	3.04
**Constant**	−3.42	0.22	249.20	1	<0.001	0.03		

**Note.** Nagelkerke R square = 0.11; classification = 81.5%.

**Table 8:** Backward binary logistic regressions cross-validated by demographic groups for predicting MV requirement.

	Sex		Age			Ethnicity	
Features	Female	Male	Young	Middle	Older	Non-Hispanic	Hispanic
Male	--	--	--	1.89 **	--	1.47 *	1.38 *
Age							
Young Adult	--	--	--	--	--	--	--
Middle	0.88	1.24	--	--	--	2.55 **	--
Older	1.72	2.02	--	--	--	8.43 **	--
BMI	--	--	--	--	--	--	--
Normal	--	0.34	0.03 **	--	1.38	1.38	--
Overweight	--	0.36	0.01 **	--	1.97	1.86	--
Obese	--	0.58	0.01 **	--	3.06 *	2.74 *	--
Race							
Black	--	--	--	--		--	--
Other	1.19	1.06	--	0.80	1.62 *	--	1.19
White	0.76 *	0.57 *	--	0.51 *	0.82	--	0.70 *
Hispanic	--	--	3.36 **	--	--	--	--
Smoke							
Never	--	--	--	--	--	--	--
Former	--	--	--	--	--	--	--
Current	--	--	--	--	--	--	--
Diabetes	1.88 **	1.48 *		2.16 *		1.49 *	1.69 **
Hypertension	1.91 **	2.35 **		2.52 *	1.66	1.99 *	2.21 **
COPD	--	--	--	--	--	--	--
Asthma	--	--	--	--	--	--	--
CKD Stages 1–4	1.61 **	1.68 **	--	--	1.95 **	1.56 *	1.82 **
CKD Stage 5	--	--	--	--	--	0.15	1.69
Heart Failure		1.49					
Cancer	--	--	--	--	--	--	--
Cardiac Arrhythmias		0.39				0.52 **	
Cerebrovascular Disease	0.35	--	--	--	--	--	0.57
Coronary Artery Disease	1.43	--	7.95 **	1.59	--	--	--
Liver Disease	--	--	8.74 **	--	--	--	--
HIV	--	--	--	--	--	--	--
Pneumonia	--	--	--	1.30	1.56 **	--	--
ARBs	0.63	0.62	--	0.48 **	0.707 **	0.51 **	1.47 **
ACEIs	0.74	0.73	--	0.59 *		--	0.67 *
Diarrhea	5.54	7.90	43.17 **	7.72 **	5.18 **	5.59 **	0.75
Dependence on Renal Dialysis	2.58		--	--	3.14 **	7.61 **	6.88 **
**R^2^**	**0.18**	**0.24**	**0.43**	**0.22**	**0.18**	**24.6**	**0.20**
**Correct Classification %**	**93.6%**	**91.5%**	**97.3%**	**94.1%**	**89.7%**	**92.4%**	**92.7%**

**Note.** Only variables that were identified as key predictors are reported for each model. Unique variance; * *p* < 0.05; ** *p* < 0.01. ‘--' in the table indicates the features not retained by the model for the sub-class.

**Table 9:** Backward binary logistic regressions cross-validated by demographic groups for predicting ICU admissions.

	Sex		Age				Ethnicity	
Features	Female	Male	Young	Middle	Older		Non-Hispanic	Hispanic
Male	--	--	--	1.87 *	1.91 *		1.75 **	1.39 **
Age								
Young Adult	--	--	--	--	--		--	--
Middle	0.89	1.26	--	--	--		2.08	0.92
Older	1.71	2.11	--	--	--		5.6 **	1.29
BMI								
Normal	--	0.29 *	0.02 **	--	--		--	--
Overweight	--	0.31	0.01 **	--	--		--	--
Obese	--	0.50	0.01 **	--	--		--	--
Race								
Black	--	--	--	--	--		--	--
Other	--	1.07	--	1.32	1.32		--	1.69 *
White	--	0.57 *	--	0.65	0.65		--	0.69 *
Hispanic	--	--	2.89 *	1.23	1.23 *		--	--
Smoke								
Never	--	--	--	--	--		--	--
Former	--	--	--	--	--		--	--
Current	--	--	--	--	--		--	--
Diabetes	1.91 *	1.47 *	--	2.42 *	2.41 **	2.08 *	1.76 **	
Hypertension	1.84 *	2.37 *	3.01 *	2.32 *	2.32 **	--	2.39 **	
COPD	--	0.65	--	--			1.76*	--
Asthma	--	--	--	--	--		--	1.78 *
CKD Stages 1–4	1.60 *	1.71 *	10.42 *	1.70 *	1.70 *		1.22	1.56 *
CKD Stage 5	--	--	6.26 *	--	--		0.15 *	--
Heart Failure	--	1.62 *	3.09 *	2.24 *	--		--	1.39 *
Cancer	--	--	--	--	--		--	--
Cardiac Arrhythmias	--	0.39	--	--	--		0.53 *	--
Cerebrovascular Disease	0.39 *	--	--	0.47 *	0.47		0.52	--
Coronary Artery Disease	1.40	--	--	--	--		1.31	--
Liver Disease	--	--	3.09*	--	--		--	--
HIV	--	--	--	--	--		--	--
Pneumonia	--	1.73	1.69	--	--		1.26	1.24 *
ARBs	0.65 *	0.62 *	0.44	0.52 *	0.51 *		--	0.66 *
ACEIs	0.73 *	0.73	0.23	0.76	0.76		0.46 **	0.69 *
Diarrhea	5.56 *	7.93 **	41.46 **	10.13 **	10.13 **	7.86 **	9.28 **	
Dependence on Renal Dialysis	2.55 *	--	--	--	--		6.39 **	--
**R^2^**	**0.18**	**0.24**	**0.46**	**0.48**	**0.27**		**0.29**	**0.26**
**Correct Classification%**	**93.6%**	**91.5%**	**95.9%**	**95.7%**	**97.1%**		**89.6%**	**89.8%**

**Note.** Only variables that were identified as key predictors are reported for each model. Unique variance; * *p* < 0.05; ** *p* < 0.01. ‘--' in the table indicates the features not retained by the model for the sub-class.

**Table 10:** Backward binary logistic regressions cross-validated by demographic groups for predicting IMCU admissions.

	Sex		Age			Ethnicity	
Features	Female	Male	Young	Middle	Older	Non-Hispanic	Hispanic
Male	--	--	--	1.64 **	1.25 *	1.40 *	1.38 *
Age							
Young Adult	--	--	--	--	--	--	--
Middle	0.86	--	--	--	--	0.85	1.16
Older	1.44	--	--	--	--	1.32	1.46
BMI	--	--	--	--	--	--	--
Normal	1.52	--	--	--	1.58	3.14	--
Overweight	2.08	--	--	--	2.07 *	5.21	--
Obese	2.32 *	--	--	--	2.16 *	5.39	--
Race							
Black	--	--	--	--	--	--	--
Other	--	--	--	--	1.24 *	--	1.12
White	--	--	--	--	0.89	--	0.91
Hispanic	--	--	--	--	--	--	--
Smoke							
Never	--	--	--	--	--	--	--
Former	--	--	0.00	--	--	--	--
Current	--	--	2.49 *	--	--	--	--
Diabetes	1.35 **	1.36 **	4.46 **	1.45 **	--	1.63 **	1.25 *
Hypertension	1.53 **	2.14 **	0.81 *	1.94 **	1.62 **	1.89 **	1.94 *
COPD	1.34 *	1.51 **	--	1.59 *	1.36 *	1.66 **	1.29 *
Asthma	--	--	2.79	--	--	--	--
CKD Stages 1–4	--	1.29 *	3.48 *	1.56 *	--	1.66 **	--
CKD Stage 5	--	--	0.42	--	--	--	--
Heart Failure	1.36 *	1.3 *	0.52	1.44 *	1.36 *	--	1.41 *
Cancer	--	--	--	1.50	--	--	0.78
Cardiac Arrhythmias	--	0.72 *	--	0.68	--	--	--
Cerebrovascular Disease	--	--	--	--	--	--	--
Coronary Artery Disease	--	--	--	--	--	--	--
Liver Disease	--	--	--	--	--	--	--
HIV	--	--	--	--	--	--	--
Pneumonia	1.42 **	1.28 *	2.25 *	1.2 *	1.35 **	1.40	1.32 *
ARBs	--	--	4.21 *	--	0.81 *	0.79	--
ACEIs	--	0.72 *	--	0.79 **	0.74 *	0.76	0.79 *
Diarrhea	2.88 **	2.42 *	2.93 *	2.81 **	2.30 **	2.82 **	2.52 *
Dependence on Renal Dialysis	0.51 *	--	--	--	--	--	0.76
**R^2^**	**0.11**	**0.09**	**0.23**	**0.11**	**0.07**	**0.15**	**0.09**
**Correct Classification %**	**83.7%**	**78.9%**	**90.5%**	**83.8%**	**76.4%**	**82.10**	**81.1**

**Note.** Only variables that were identified as key predictors are reported for each model. Unique variance; * *p* < 0.05; ** *p* < 0.01. ‘--' in the table indicates the features not retained by the model for the sub-class.

## Data Availability

This dataset is provided by the South Florida Memorial Healthcare System for analysis. We cannot make this publicly available without their permission. You can contact the corresponding authors to request permission to access the data from the contact at South Florida Memorial Healthcare System.

## References

[1] Miller I.F., Becker A.D., Grenfell B.T., Metcalf C.J.E. (2020). Disease and healthcare burden of COVID-19 in the United States. Nat. Med..

[2] Worldometer. COVID-19 Coronavirus Pandemic, 2023. World Health Organization (WHO). COVID-19 Weekly Epidemiological Update 2023. https://www.worldometers.info/coronavirus/.

[3] Richardson S., Hirsch J.S., Narasimhan M., Crawford J.M., McGinn T., Davidson K.W., the Northwell COVID-19 Research Consortium (2020). Presenting Characteristics, Comorbidities, and Outcomes among 5700 Patients H Hospitalized with COVID-19 in the New York City Area. JAMA.

[4] Meille G., Decker S.L., Owens P.L., Selden T.M. (2023). COVID-19 admission rates and changes in US hospital inpatient and intensive care unit occupancy. JAMA Health Forum.

[5] Ranney M.L., Griffeth V., Jha A.K. (2020). Critical supply shortages—The need for ventilators and personal protective equipment during the COVID-19 pandemic. N. Engl. J. Med..

[6] Schwab P., DuMont Schütte A., Dietz B., Bauer S. (2020). Clinical predictive models for COVID-19: Systematic study. J. Med. Internet Res..

[7] Bhatraju P.K., Ghassemieh B.J., Nichols M., Kim R., Jerome K.R., Nalla A.K., Greninger A.L., Pipavath S., Wurfel M.M., Evans L. (2020). COVID-19 in critically ill patients in the Seattle region—Case series. N. Engl. J. Med..

[8] Cummings M.J., Baldwin M.R., Abrams D., Jacobson S.D., Meyer B.J., Balough E.M., Aaron J.G., Claassen J., Rabbani L.E., Hastie J. (2020). Epidemiology, clinical course, and outcomes of critically ill adults with COVID-19 in New York City: A prospective cohort study. Lancet.

[9] Gupta S., Hayek S.S., Wang W., Chan L., Mathews K.S., Melamed M.L., Brenner S.K., Leonberg-Yoo A., Schenck E.J., Radbel J. (2020). Factors associated with death in critically ill patients with coronavirus disease 2019 in the US. JAMA Intern. Med..

[10] Zhang A., Xing L., Zou J., Wu J.C. (2022). Shifting machine learning for healthcare from development to deployment and from models to data. Nat. Biomed. Eng..

[11] Abràmoff M.D., Lavin P.T., Birch M., Shah N., Folk J.C. (2018). Pivotal trial of an autonomous AI-based diagnostic system for detection of diabetic retinopathy in primary care offices. NPJ Digit. Med..

[12] Johnson K.B., Wei W., Weeraratne D., Frisse M.E., Misulis K., Rhee K., Zhao J., Snowdon J.L. (2021). Precision medicine, AI, and the future of personalized health care. Clin. Transl. Sci..

[13] Dixon D., Sattar H., Moros N., Kesireddy S.R., Ahsan H., Lakkimsetti M., Fatima M., Doshi D., Sadhu K., Hassan M.J. (2024). Unveiling the Influence of AI Predictive Analytics on Patient Outcomes: A Comprehensive Narrative Review. Cureus.

[14] Hilton C.B., Milinovich A., Felix C., Vakharia N., Crone T., Donovan C., Proctor A., Nazha A. (2020). Personalized predictions of patient outcomes during and after hospitalization using artificial intelligence. NPJ Digit. Med..

[15] Chen Z., Russo N.W., Miller M.M., Murphy R.X., Burmeister D.B. (2021). An observational study to develop a scoring system and model to detect risk of hospital admission due to COVID-19. J. Am. Coll. Emerg. Physicians Open.

[16] Liang W., Liang H., Ou L., Chen B., Chen A., Li C., Li Y., Guan W., Sang L., Lu J. (2020). Development and validation of a clinical risk score to predict the occurrence of critical illness in hospitalized patients with COVID-19. JAMA Intern. Med..

[17] Zhao Z., Chen A., Hou W., Graham J.M., Li H., Richman P.S., Thode H.C., Singer A.J., Duong T.Q. (2020). Prediction model and risk scores of ICU admission and mortality in COVID-19. PLoS ONE.

[18] Noy O., Coster D., Metzger M., Atar I., Shenhar-Tsarfaty S., Berliner S., Rahav G., Rogowski O., Shamir R. (2022). A machine learning model for predicting deterioration of COVID-19 inpatients. Sci. Rep..

[19] Ferrari D., Milic J., Tonelli R., Ghinelli F., Meschiari M., Volpi S., Faltoni M., Franceschi G., Iadisernia V., Yaacoub D. (2020). Machine learning in predicting respiratory failure in patients with COVID-19 pneumonia—Challenges, strengths, and opportunities in a global health emergency. PLoS ONE.

[20] Ryan C., Minc A., Caceres J., Balsalobre A., Dixit A., Ng B.K., Schmitzberger F., Syed-Abdul S., Fung C. (2021). Predicting severe outcomes in COVID-19 related illness using only patient demographics, comorbidities and symptoms. Am. J. Emerg. Med..

[21] Singh V., Kamaleswaran R., Chalfin D., Buño-Soto A., Roman J.S., Rojas-Kenney E., Molinaro R., von Sengbusch S., Hodjat P., Comaniciu D. (2021). A deep learning approach for predicting severity of COVID-19 patients using a parsimonious set of laboratory markers. iScience.

[22] Chieregato M., Frangiamore F., Morassi M., Baresi C., Nici S., Bassetti C., Bnà C., Galelli M. (2022). A hybrid machine learning/deep learning COVID-19 severity predictive model from CT images and clinical data. Sci. Rep..

[23] Li X., Ge P., Zhu J., Li H., Graham J., Singer A., Richman P.S., Duong T.Q. (2020). Deep learning prediction of likelihood of ICU admission and mortality in COVID-19 patients using clinical variables. PeerJ.

[24] Magunia H., Lederer S., Verbuecheln R., Gilot B.J., Koeppen M., Haeberle H.A., Mirakaj V., Hofmann P., Marx G., Bickenbach J. (2021). Machine learning identifies ICU outcome predictors in a multicenter COVID-19 cohort. Crit. Care.

[25] Beiser D.G., Jarou Z.J., Kassir A.A., Puskarich M.A., Vrablik M.C., Rosenman E.D., McDonald S.A., Meltzer A.C., Courtney D.M., Kabrhel C. (2021). Predicting 30-day return hospital admissions in patients with COVID-19 discharged from the emergency department: A national retrospective cohort study. J. Am. Coll. Emerg. Physicians Open.

[26] Garcia-Gutiérrez S., Esteban-Aizpiri C., Lafuente I., Barrio I., Quiros R., Quintana J.M., Uranga A. (2022). Machine learning-based model for prediction of clinical deterioration in hospitalized patients by COVID 19. Sci. Rep..

[27] Liu Q., Pang B., Li H., Zhang B., Liu Y., Lai L., Le W., Li J., Xia T., Zhang X. (2021). Machine learning models for predicting critical illness risk in hospitalized patients with COVID-19 pneumonia. J. Thorac. Dis..

[28] Purkayastha S., Xiao Y., Jiao Z., Thepumnoeysuk R., Halsey K., Wu J., Tran T.M.L., Hsieh B., Choi J.W., Wang D. (2021). Machine learning-based prediction of COVID-19 severity and progression to critical illness using CT imaging and clinical data. Korean J. Radiol..

[29] Hong W., Zhou X., Jin S., Lu Y., Pan J., Lin Q., Yang S., Xu T., Basharat Z., Zippi M. (2022). A comparison of XGBoost, random forest, and nomograph for the prediction of disease severity in patients with COVID-19 pneumonia: Implications of cytokine and immune cell profile. Front. Cell. Infect. Microbiol..

[30] Patel D., Kher V., Desai B., Lei X., Cen S., Nanda N., Gholamrezanezhad A., Duddalwar V., Varghese B., AOberai A. (2021). Machine learning based predictors for COVID-19 disease severity. Sci. Rep..

[31] Datta D., Dalmida S.G., Martinez L., Newman D., Hashemi J., Khoshgoftaar T.M., Shorten C., Sareli C., Eckardt P. (2023). Using machine learning to identify patient characteristics to predict mortality of in-patients with COVID-19 in south Florida. Front. Digit. Health.

[32] Shorten C., Cardenas E., Khoshgoftaar T.M., Hashemi J., Dalmida S.G., Newman D., Datta D., Martinez L., Sareli C., Eckard P. Exploring Language-Interfaced Fine-Tuning for COVID-19 Patient Survival Classification. Proceedings of the 2022 IEEE 34th International Conference on Tools with Artificial Intelligence (ICTAI).

[33] Shorten C., Khoshgoftaar T.M., Hashemi J., Dalmida S.G., Newman D., Datta D., Martinez L., Sareli C., Eckard P. Predicting the Severity of COVID-19 Respiratory Illness with Deep Learning. Proceedings of the International FLAIRS Conference Proceedings.

[34] Bennett D.A. (2001). How can I deal with missing data in my study?. Aust. N. Z. J. Public Health.

[35] Statsenko Y., Al Zahmi F., Habuza T., Almansoori T.M., Smetanina D., Simiyu G.L., Gorkom K.N.-V., Ljubisavljevic M., Awawdeh R., Elshekhali H. (2022). Impact of Age and Sex on COVID-19 Severity Assessed From Radiologic and Clinical Findings. Front. Cell. Infect. Microbiol..

[36] Romaine D.S., Randall O.S. (2005). The Encyclopedia of the Heart and Heart Disease.

[37] Hancock J.T., Khoshgoftaar T.M. (2020). Survey on categorical data for neural networks. J. Big Data.

[38] Kubinger K.D. (2003). On artificial results due to using factor analysis for dichotomous variables. Psychol. Sci..

[39] Deb D., Smith R.M. (2021). Application of Random Forest and SHAP Tree Explainer in Exploring Spatial (In) Justice to Aid Urban Planning. ISPRS Int. J. Geo Inf..

[40] Pedregosa F., Varoquaux G., Gramfort A., Michel V., Thirion B., Grisel O., Blondel M., Prettenhofer P., Weiss R., Dubourg V. (2011). Scikit-learn: Machine learning in Python. J. Mach. Learn. Res..

[41] Chawla N.V., Bowyer K.W., Hall L.O., Kegelmeyer W.P. (2002). SMOTE: Synthetic minority over-sampling technique. J. Artif. Intell. Res..

[42] Abd El-Raheem G.O.H., Mohamed D.S.I., Yousif M.A.A., Elamin H.E.S. (2021). Characteristics and severity of COVID-19 among Sudanese patients during the waves of the pandemic. Sci. Afr..

[43] Hosmer D.W., Lemeshow S., Sturdivant R.X. (2013). Applied Logistic Regression.

[44] Steyerberg E.W., Eijkemans M.J., Habbema J.F. (2018). Stepwise selection in small data sets: A simulation study of bias in logistic regression analysis. J. Clin. Epidemiol..

[45] Harrell F.E. (2015). Regression Modeling Strategies: With Applications to Linear Models, Logistic Regression, and Survival Analysis.

[46] Ambler G., Brady A.R., Royston P. (2002). Simplifying a prognostic model: A simulation study based on clinical data. Stat. Med..

[47] Wang H., Li R., Tsai C.L. (2007). Tuning parameter selectors for the smoothly clipped absolute deviation method. Biometrika.

[48] Hicks S.A., Strümke I., Thambawita V., Hammou M., Riegler M.A., Halvorsen P., Parasa S. (2022). On evaluation metrics for medical applications of artificial intelligence. Sci. Rep..

[49] Hamida S., El Gannour O., Cherradi B., Ouajji H., Raihani A. Optimization of Machine Learning Algorithms Hyper-Parameters for Improving the Prediction of Patients Infected with COVID-19. Proceedings of the 2020 IEEE 2nd international conference on electronics, control, optimization and computer science (ICECOCS).

[50] Sah S., Surendiran B., Dhanalakshmi R., Yamin M. (2023). COVID-19 cases prediction using SARIMAX Model by tuning hyperparameter through grid search cross-validation approach. Expert Syst..

[51] Liu B., Udell M. (2020). Impact of accuracy on model interpretations. arXiv.

[52] Ishwaran H. (2015). The effect of splitting on random forests. Mach. Learn..

[53] Kim Y., Kim Y. (2022). Explainable heat-related mortality with random forest and SHapley additive exPlanations (SHAP) models. Sustain. Cities Soc..

[54] Zhai B., Perez-Pozuelo I., Clifton E.A., Palotti J., Guan Y. (2020). Making sense of sleep: Multimodal sleep stage classification in a large, diverse population using movement and cardiac sensing. ACM Interact. Mob. Wearable Ubiquitous Technol..

[55] Rodríguez-Pérez R., Bajorath J. (2019). Interpretation of compound activity predictions from complex machine learning models using local approximations and shapley values. J. Med. Chem..

[56] Altmann A., Toloşi L., Sander O., Lengauer T. (2010). Permutation importance: A corrected feature importance measure. Bioinformatics.

[57] Van Lissa C.J., Stroebe W., Leander N.P., Agostini M., Draws T., Grygoryshyn A., Gützgow B., Kreienkamp J., Vetter C.S., Abakoumkin G. (2022). Using machine learning to identify important predictors of COVID-19 infection prevention behaviors during the early phase of the pandemic. Patterns.

[58] Moncada-Torres A., van Maaren M.C., Hendriks M.P., Siesling S., Geleijnse G. (2021). Explainable machine learning can outperform cox regression predictions and provide insights in breast cancer survival. Sci. Rep..

[59] Passarelli-Araujo H., Passarelli-Araujo H., Urbano M.R., Pescim R.R. (2022). Machine learning and comorbidity network analysis for hospitalized patients with COVID-19 in a city in southern Brazil. Smart Health.

[60] Batunacun, Wieland R., Lakes T., Nendel C. (2020). Using SHAP to interpret XGBoost predictions of grassland degradation in Xilingol, China. Geosci. Mod. Dev. Discuss..

[61] Gómez-Ramírez J., Ávila-Villanueva M., Fernández-Blázquez M.Á. (2020). Selecting the most important self-assessed features for predicting conversion to mild cognitive impairment with random forest and permutation-based methods. Sci. Rep..

[62] Huang X., Marques-Silva J. (2024). On the failings of Shapley values for explainability. Int. J. Approx. Reason..

[63] Kumar I.E., Venkatasubramanian S., Scheidegger C., Friedler S. Problems with Shapley-Value-Based Explanations as Feature Importance Measures. Proceedings of the International Conference on Machine Learning.

[64] Molnar C., Freiesleben T., König G., Herbinger J., Reisinger T., Casalicchio G., Wright M.N., Bischl B. (2023). Relating the Partial Dependence Plot and Permutation Feature Importance to the Data Generating Process. Proceedings of the World Conference on Explainable Artificial Intelligence.

[65] Nohara Y., Inoguchi T., Nojiri C., Nakashima N. (2022). Explanation of Machine Learning Models of Colon Cancer Using SHAP Considering Interaction Effects. arXiv.

[66] Lundberg S.M., Erion G., Chen H., DeGrave A., Prutkin J.M., Nair B., Katz R., Himmelfarb J., Bansal N., Lee S.-I. (2020). From local explanations to global understanding with explainable AI for trees. Nat. Mach. Intell..

[67] Qiu W., Chen H., Dincer A.B., Lundberg S., Kaeberlein M., Lee S.I. (2022). Interpretable machine learning prediction of all-cause mortality. Commun. Med..

[68] Molani S., Hernandez P.V., Roper R.T., Duvvuri V.R., Baumgartner A.M., Goldman J.D., Ertekin-Taner N., Funk C.C., Price N.D., Rappaport N. (2022). Risk factors for severe COVID-19 differ by age for hospitalized adults. Sci. Rep..

[69] Ebinger J.E., Achamallah N., Ji H., Claggett B.L., Sun N., Botting P., Nguyen T.-T., Luong E., Kim E.H., Park E. (2020). Pre-existing traits associated with COVID-19 illness severity. PLoS ONE.

[70] Şenkal N., Meral R., Medetalibeyoğlu A., Konyaoğlu H., Kose M., Tukek T. (2020). Association between chronic ACE inhibitor exposure and decreased odds of severe disease in patients with COVID-19. Anatol. J. Cardiol..

[71] Zhang X., Cai H., Hu J., Lian J., Gu J., Zhang S., Ye C., Lu Y., Jin C., Yu G. (2020). Epidemiological, clinical characteristics of cases of SARS-CoV-2 infection with abnormal imaging findings. Int. J. Infect. Dis..

[72] Ge L., Meng Y., Ma W., Mu J. (2024). A retrospective prognostic evaluation using unsupervised learning in the treatment of COVID-19 patients with hypertension treated with ACEI/ARB drugs. PeerJ.

[73] Wu H., Ruan W., Wang J., Zheng D., Liu B., Geng Y., Chai X., Chen J., Li K., Li S. (2021). Interpretable machine learning for covid-19: An empirical study on severity prediction task. IEEE Trans. Artif. Intell..

[74] Ueda D., Kakinuma T., Fujita S., Kamagata K., Fushimi Y., Ito R., Matsui Y., Nozaki T., Nakaura T., Fujima N. (2024). Fairness of artificial intelligence in healthcare: Review and recommendations. Jpn. J. Radiol..

[75] Ghassemi M., Naumann T., Schulam P., Beam A.L., Chen I.Y., Ranganath R. (2020). A review of challenges and opportunities in machine learning for health. AMIA Summits Transl. Sci. Proc..

[76] Kelly C.J., Karthikesalingam A., Suleyman M., Corrado G., King D. (2019). Key challenges for delivering clinical impact with artificial intelligence. BMC Med..

[77] Cohen J.P., Cao T., Viviano J.D., Huang C.-W., Fralick M., Ghassemi M., Mamdani M., Greiner R., Bengio Y. (2021). Problems in the deployment of machine-learned models in health care. Can. Med. Assoc. J..

[78] Laatifi M., Douzi S., Bouklouz A., Ezzine H., Jaafari J., Zaid Y., El Ouahidi B., Naciri M. (2022). Machine learning approaches in COVID-19 severity risk prediction in Morocco. J. Big Data.

[79] Khadem H., Nemat H., Elliott J., Benaissa M. (2022). Interpretable machine learning for inpatient COVID-19 mortality risk assessments: Diabetes mellitus exclusive interplay. Sensors.

